# Bioengineered *Escherichia coli* Nissle 1917 for tumour‐targeting therapy

**DOI:** 10.1111/1751-7915.13523

**Published:** 2019-12-21

**Authors:** Xiaoli Yu, Changsen Lin, Jing Yu, Qingsheng Qi, Qian Wang

**Affiliations:** ^1^ School of Public Health and Management Weifang Medical University Weifang 261053 Shandong China; ^2^ State Key Laboratory of Microbial Technology National Glycoengineering Research Center Shandong University Qingdao 266237 Shandong China; ^3^ Affiliated Hospital of Shandong University of Traditional Chinese Medicine Jinan 250014 Shandong China

## Abstract

Bacterial vectors, as microscopic living ‘robotic factories’, can be reprogrammed into microscopic living ‘robotic factories’, using a top‐down bioengineering approach to produce and deliver anticancer agents. Most of the current research has focused on bacterial species such as *Salmonella typhimurium* or *Clostridium novyi*. However, *Escherichia coli* Nissle 1917 (EcN) is another promising candidate with probiotic properties. EcN offers increased applicability for cancer treatment with the development of new molecular biology and complete genome sequencing techniques. In this review, we discuss the genetics and physical properties of EcN. We also summarize and analyse recent studies regarding tumour therapy mediated by EcN. Many challenges remain in the development of more promising strategies for combatting cancer with EcN.

## Graphical abstract

*Escherichia coli* Nissle 1917 (EcN) is a promising candidate with probiotic properties for cancer treatment. This review discuss the genetics and physical properties of EcN, and summarize recent studies regarding tumour therapy mediated by EcN.

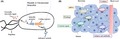

## Introduction

Bacteria may be considered programmable ‘robot factories’ that specifically target tumours, and they have unique capabilities that make them well‐suited to be ideal anticancer agents (Forbes, 2010). Recently, the mechanism of action and antitumour effects of bacteria on tumour cells has been studied (Maeda, 2013; Zhang and Forbes, 2015; Zhou *et al.*, 2018). Bacteria exhibit intrinsic antitumour activity, because they express chemotactic receptors, which direct chemotaxis towards molecular signals in the tumour microenvironment. They are also equipped with flagella, which facilitates tissue penetration (Grozdanov *et al.*, 2004; Reister *et al.*, 2014). They can migrate and accumulate far from the vasculature. They may also be engineered to sense and respond to the tumour microenvironment resulting in innate and adaptive antitumour immune responses (Zhou *et al.*, 2018). However, the antitumour effect of bacteria within tumours is generally weak, and different bacteria and treatment strategies have been developed to enhance their antitumour effect (Piñero‐Lambea *et al.*, 2015b). In addition, some bacteria such as *Escherichia coli* are currently bioengineered using a variety of molecular tools to produce biologically active molecules.

Many studies have focused on the reproductive features of bacteria in combination with their capacity to produce living therapeutics. As a next‐generation therapy, these tiny living factories may decrease production costs, reduce side‐effects, require smaller doses of biological compound and produce more compounds (Pedrolli *et al.*, 2019).

**Figure 1:**
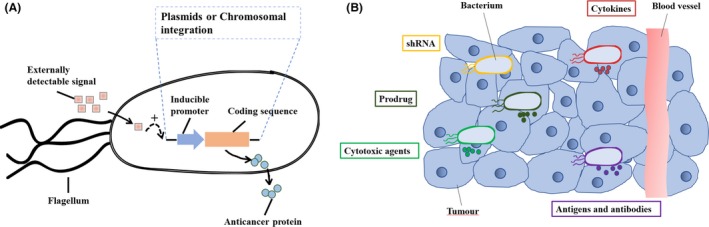
Engineered bacteria for cancer therapy. A. The bacterial cell with various inducible systems for payload expression. B. The bacterial accumulation and replication within solid tumours enabling localized expression of anticancer agents

Thus far, bacteria such as *Clostridium* sp. (Agrawal *et al.*, 2004), *Bifidobacterium* sp. (Sasaki *et al.*, 2006), *Salmonella* sp. (Mengesha *et al.*, 2006) and *Escherichia* sp. (Yu *et al.*, 2004) have been engineered to deliver RNA (Yang *et al.*, 2008), prodrugs (Hedley *et al.*, 2007), cytotoxic agents (Ryan *et al.*, 2009), cytokines (Loeffler *et al.*, 2008), antigens (Nishikawa *et al.*, 2006) and antibodies (Groot *et al.*, 2007). All of these bacteria have been genetically modified to increase the effectiveness of anticancer agents (Fig. 1). A straightforward approach is to engineer bacteria to express proteins such as bacterial toxins to eradicate cancer cells. Bacterial toxins, such as cytolysin A, affect the mammalian cell membranes and induce apoptosis (Jiang *et al.*, 2010). This strategy requires bacterial vectors that specifically target cancer cells or vectors with inducible promoters for better control of gene expression to avoid toxicity to normal tissues (Loessner *et al.*, 2009). Another common strategy is to engineer these bacteria to express prodrug‐converting enzymes. The major advantage of these enzymes is that the resulting cytotoxic products can permeate the cell membrane and diffuse farther inside the solid tumour (Lehouritis *et al.*, 2016).

Bacteria can be genetically engineered in a variety of ways to create a versatile living platform that can deliver a therapeutic payload based on clinical needs. For effective cancer therapy, the initial concern is to select an appropriate bacterial strain (Pedrolli *et al.*, 2019). The facultative anaerobe, *Salmonella typhimurium*, has been widely studied and engineered to improve its tumour‐targeting ability. It has even been applied in human clinical trials (Toso *et al.*, 2002; Thamm *et al.*, 2005). However, *Salmonella*, as well as other toxic strains including *Clostridium novyi* (Dang *et al.*, 2001) and *Listeria monocytogenes* (Freitag *et al.*, 1993), should be modified to improve its safety profile. Moreover, the effect of *Salmonella*‐mediated therapy for cancer is smaller than that of virulence‐attenuated *Shigella flexneri* 21 SC602 and other *E. coli* strains (Stritzker *et al.*, 2007). Among these bacteria, non‐pathogenic *E. coli* strains such as K‐12 and Nissle 1917 also exhibit tumour‐targeting activity (Stritzker *et al.*, 2007; Weibel *et al.*, 2008; Piñero‐Lambea *et al.*, 2015a). *E. coli* Nissle 1917 (EcN) is probably a better choice with its probiotic potential, and it has been widely used and combined into living therapeutics. EcN can be genetically engineered to act as a living therapeutic to treat solid cancers (Singh *et al.*, 2017; Chua *et al.*, 2017). These findings provide a rationale for EcN as a promising probiotic for cancer. This review covers recent methods to engineer EcN for cancer treatment and provides a primary resource for scientists choosing EcN to create new living therapeutics.

## Characteristics of *E. coli* Nissle 1917

*Escherichia coli* Nissle 1917 (EcN) is a Gram‐negative probiotic, originally isolated by Dr. Alfred Nissle during World War I (Nissle, 1918; Nissle, 1925). EcN is serum‐sensitive and does not produce any enterotoxins or cytotoxins associated with pathogenic *E. coli* strains (Sonnenborn and Schulze, 2009). It has been licensed as a pharmaceutical for the treatment of diseases such as diarrhoea and colitis ulcerosa (Kruis *et al.*, 2004). Along with the new finding of its biological function, the genetics of EcN has been extensively characterized. It was discovered that EcN harbours two cryptic plasmids, named pMUT1 and pMUT2. Both plasmids have been completely sequenced and shown to be genetically stable and non‐transferable (Blum‐Oehler *et al.*, 2003; Sonnenborn and Schulze, 2009). A plasmid‐free variant of EcN was shown not to be functionally different from the wild‐type EcN strain and may be used as a live vector for recombinant plasmids, based on pMUT1 and pMUT2 (Oswald, 2006).

Analysis of the EcN genome structure has further revealed that the lack of defined virulence factors, such as alpha‐hemolysin, P‐fimbrial adhesins and the semi‐rough lipopolysaccharide phenotype combined with the expression of fitness factors, such as microcins, adhesins and iron uptake systems, may contribute to its probiotic character (Grozdanov *et al.*, 2004). Genomic islands, which synthesize ‘fitness factors’, are located within the EcN chromosome. These islands increase bacterial fitness and are crucial to their ability to colonize a host (Hacker and Carniel, 2001). To further elucidate the molecular basis for EcN’s probiotic nature, the genomic peculiarity of coding sequences and reconstructed metabolic network, inferred from raw genome data, was studied (Sun *et al.*, 2005). In 2014, Reister *et al. *(2014) reported EcN’s complete and annotated genomic sequence (Genbank accession number: CP007799) and identified the genes and their products essential for its probiotic nature. The size of the EcN genome is 5 441 200 bp, and it contains 5324 predicted genes. Metabolomic studies have revealed that EcN displays substantial metabolic differences as compared with phylogenetically similar pathogenic *E. coli* strains, thus revealing its potent probiotic characteristics (van der Hooft *et al.*, 2019). Understanding the genetics and characteristics of EcN will lead to engineering methods to effectively manipulate EcN.

## The mechanisms of EcN as an antitumour agent

Bacteria use complex mechanisms to target tumours. Different bacterial species share unique intrinsic mechanisms to eliminate cancer. The mechanisms of accumulation within tumours differ and rely on oxygen tolerance. EcN, as a facultative anaerobe, may utilize complex mechanisms to target tumours. The EcN serotype O6:K5:H1 is an excellent example of bacterial genome evolution within the pathogenic *E. coli* serotype O6 lineage (Behnsen *et al.*, 2013). It is devoid of prominent virulence genes and displays fitness factors that contribute to its colonization efficiency and survival within the host (Sanders, 2003). Moreover, the serum‐sensitive LPS of the EcN membrane ensures the quick elimination of the strain from normal organs, and it is free of immunotoxic side‐effects in patients (Grozdanov *et al.*, 2002). These striking features likely confer protection from clearance by the host immune system.

Another characteristic of EcN is the extracellular K5 capsule, which is important for adhesion and colonization (Burns and Hull, 1998). Bacteria are reported to proliferate preferentially within solid tumours (Pawelek *et al.*, 2003), and this feature probably promotes the targeting of EcN to the tumour, resulting in preferential growth within the tumour microenvironment. It has also been shown that EcN contributes to reduced inflammation by downregulating the expansion of newly recruited T cells into the mucosa (Sturm *et al.*, 2005). This indicates that one mechanism of EcN‐mediated tumour targeting occurs following inflammation.

However, different bacteria in different microenvironments adopt distinct antitumour mechanisms. The specific mechanism may differ depending on which bacterial species is used, tumour type and dynamics of the bacteria‐host interaction (Zhou *et al.*, 2018). The mechanisms of EcN interaction in different environments facilitate accumulation within tumours.

## Exploration of EcN for tumour‐targeting therapy

Many biological tools such as Red/ET recombination and CRISPR‐Cas9 are currently available for engineering *E. coli* (Jiang *et al.*, 2013). Based on its ‘tumour‐finding’ nature, *E. coli* is a programmable delivery vehicle that may be designed to carry multiple genes for therapeutic or diagnostic cancer agents (Pedrolli *et al.*, 2019). EcN is one promising *E. coli* strain that can be engineered systemically, resulting in exclusive tumour colonization in live mice (Stritzker *et al.*, 2007). Positron emission tomography (PET) and optical imaging have been used to monitor the tumour‐targeting activity of EcN (Brader *et al.*, 2008). Genome sequence analysis has revealed EcN to be a novel bioengineered probiotic with several unique properties such as (i) interaction with the host immune system (Sturm *et al.*, 2005), (ii) antimicrobial activity through secretion of microcins and bacteriocins (Sassone‐Corsi *et al.*, 2016), and (iii) formation of biofilms resulting in the production of defensins (Lasaro *et al.*, 2009). A key step in the engineering of living therapeutics is to choose a suitable chassis, preferably a probiotic with optimal pharmacologic properties (Claesen and Fischbach, 2015). Due to its unique function and high versatility, EcN has provided new opportunities for next‐generation therapeutic and probiotic therapies. Using these modalities, researchers have observed reduced tumour volume, increased survival and eradication of metastatic disease in animal models, while avoiding damage to healthy cells (Table 1).

**Table 1:** Current summary of engineered EcN for clinical cancer exploration.

Effector classes	Effectors or targets	Cancer type	References
Cytotoxic agents	Azurin	Mouse B16 melanoma and human 4T1 breast tumours	Zhang *et al. *(2012)
	Colibactin, glidobactin and luminmide	Human U‐2 OS osteosarcoma cells	Li *et al. *(2019)
	p53	Human hepatoma SMMC‐7721 cells	He *et al. *(2017)
Synthetic gene circuit	Genomic luxCDABE cassette and lacZ vector to develop a diagnostic platform PROP‐Z	Metastatic murine colorectal cells	Danino *et al. *(2015)
Tumour stroma targeting	Tumstatin	Mouse B16 melanoma cells	He *et al. *(2017)
		Human hepatoma SMMC‐7721 cells	He *et al. *(2019)
Prodrug‐converting enzymes	Myrosinase	Murine, human and colorectal adenocarcinoma cell lines	Ho *et al. *(2018)
Prodrugs	CB1954, 5‐FC and Fludarabine phosphate	Mouse CT26 colon cells	Lehouritis *et al*. (2016)

### Expression of therapeutic proteins in EcN

The major strategy of EcN‐mediated tumour therapy is to transform plasmids carrying gene expression cassettes to direct the expression of therapeutic proteins (Table 1). For example, the azurin protein was constitutively expressed to inhibit tumours (Zhang *et al.*, 2012). In this study, BALB/c mice, bearing orthotopic B16 melanoma or 4T1 breast tumours, were administered PBS, EcN and its variants by intravenous (i.v.) injection at a dose of 2 × 10^7^ CFU per mouse. Tumour growth and pulmonary metastasis were efficiently suppressed by azurin release and the resulting inflammatory response without significant toxicity. For the detection of liver metastasis in urine samples, Danino *et al. *(2015) engineered EcN to carry a genomic luxCDABE cassette containing a high‐expression lacZ vector. This was used to develop a diagnostic platform, PROP‐Z. A murine model of colorectal cancer metastases was used in which spleens from immunocompetent BALB/c mice were surgically injected with metastatic murine colorectal cells (MC26‐LucF). After PROP‐Z was delivered orally, EcN rapidly (within 24 h) translocated across the gastrointestinal tract and specifically colonized within the metastatic tumours present in the liver, but not within healthy organs or fibrotic liver tissue. PROP‐Z expressed high levels of the enzyme lacZ, which cleaves a substrate to produce a small molecule that can be detected in urine. EcN was also selected as a vector to specifically express Tum‐5, which is a suitable tumour‐specific angiogenesis inhibitor. Tum‐5 was expressed in EcN under control by the oxygen‐dependent promoter of the haemoglobin gene (*vhb*) from *Vitreoscilla*. The colonization of EcN (Tum‐5) was investigated in C57BL/6 mice bearing B16 melanoma at different time points following i.p. injection of 5×10^6^ CFU/100 µL EcN. Tumour growth and angiogenesis were inhibited by upregulation of Tum‐5 expression (He *et al.*, 2017). In addition, biosynthetic gene clusters encoding cytotoxic compounds such as colibactin, glidobactin and luminmide were introduced into EcN. EcN and its variants were administered by i.v. injection into female NMRI nude mice bearing UT‐SCC‐5 human head and neck squamous tumours at a dose of 1 × 10^7^ CFU per mouse. The colibactin/glidobactins/lumimides‐expressing EcN exhibited significant cytotoxic activity and suppressed tumour growth (Li *et al.*, 2019). The anticancer protein p53 and the anti‐angiogenic factor Tum‐5 were constructed as bifunctional proteins and delivered to solid tumours using EcN (He *et al.*, 2019). In this study, the SMMC‐7721 tumour‐bearing BALB/c nude mice were i.v. injected with EcN and its variants at a dose of 5 × 10^6^ CFU/100 µL. Treatment with the engineered bacteria led to significant inhibitory effects on the growth of orthotopic hepatoma tumours without notable toxicity.

The expression of therapeutic proteins in EcN can successfully regress tumours. However, the accumulation of therapeutic proteins should be synthesized at sufficient concentrations to induce a therapeutic effect, but not high enough to cause systemic toxicity.

### Expression of prodrug‐converting enzymes in EcN

Another strategy is to express prodrug‐converting enzymes that can metabolize their corresponding prodrug substrates and convert them into cytotoxic products, thus generating a potent bystander effect (a therapeutic effect on cells that is not influenced by bacteria) (Table 1). Ho *et al. *(2018) selected alanine‐deficient EcN to co‐express *INP‐HlpA* (Protein HlpA from *Streptococcus gallolyticus* with an INP tag) and *YebF‐I1* (Myrosinase from *Armoracia rusticana* with a YebF‐secretion tag) using constitutive promoters. Engineered EcN was orally administered and bound specifically to heparan sulfate proteoglycan on colorectal cancer cells. As a result, secreted myrosinase converted dietary glucosinolate to sulforaphane, an organic molecule with anticancer activity. This combinatorial approach led to an almost complete inhibition of proliferation in murine and human colorectal adenocarcinoma cell lines *in vitro*. Tumour regression and decreased tumour formation were observed in a murine CRC model fed with the engineered living therapeutic. The efficiency of this strategy relies on the continued high‐level expression of prodrug‐converting enzymes, resulting in the sustained tumour colonization by the bacterial vector. Moreover, EcN has the ability to activate numerous prodrugs and is resistant to prodrug toxicity. Therefore, it was selected to activate multiple prodrugs such as CB1954, 5‐FC and Fludarabine phosphate without genetic modification (Lehouritis *et al.*, 2016). In this study, BALB/c mice bearing subcutaneous CT26 flank tumours were colonized with EcN (5 × 10^5^ CFU/50 µL) by intratumoural injection. CB1954, 5‐FC or a combination of both drugs was also administered i.p. into mice on the same day. The combined use of EcN and prodrugs led to a significant reduction in tumour growth, indicating their potential role in solid tumour treatment.

### Engineering of EcN‐derived minicells

In addition to the delivery of EcN payloads, EcN‐derived minicells and bacterial ghosts (BGs) may be modified and filled with tumour‐targeted drugs. MacDiarmid *et al. *(2007) reported that minicells are nanosized forms of bacteria and contain the same cytoplasmic components as their parental bacteria with the exception of chromosomal DNA. The lack of a genome in the minicells results in a loss of proliferation, but minicells maintain other characteristics inherited from their parental bacteria. Minicells have been used for targeted delivery of siRNA or chemotherapeutic drugs into tumours. These drug‐loaded minicells, which may be modified with antibodies to receptors on cancer cells, can target tumours and release anticancer drugs (MacDiarmid *et al.*, 2009; MacDiarmid *et al.*, 2016). Additionally, Zhang *et al. *(2018) found that pHLIP‐mosaic minicell treatment resulted in a significant regression of orthotopic breast tumours in a BALB/c mice model along with high biocompatibility and low toxicity. The EcN‐derived minicells were produced by knocking out the *minCD* gene and enhancing *minE* expression. A pH (low) insertion peptide (pHLIP) was displayed on the membrane surface through the Lpp‐OmpA’ protein display system to increase targeting efficiency. Then, the EcN‐derived minicells displaying pHLIP were directly extracted from the fermentation broth and loaded with doxorubicin (DOX). EcN was also used as a bacterial carrier to immobilize amphiphilic copolymers through acid‐labile linkers (Xie *et al.*, 2018). The released copolymers were self‐assembled into micelles. These hybrid micelles released both doxorubicin and α‐tocopheryl succinate, which resulted in synergistic antitumour activity in 4T1 tumour‐bearing mice. These studies provide novel strategies for constructing delivery systems by genetically modifying EcN‐derived minicells and utilizing biomaterials that have the ability to penetrate tumours.

### Engineering of EcN BGs

Bacterial ghosts are considered to be empty and intact non‐living bacterial cell envelopes that may be used as a compound delivery system (Langemann *et al.*, 2010; Kraśko *et al.*, 2017). BGs are devoid of any cytoplasmic content but retain natural outer surface composition. Based on these characteristics, BGs have excellent carrier capacity and immunogenicity, and retain the original targeting functions of parental bacteria (Ganeshpurkar *et al.*, 2014). EcN BGs have been prepared by fusion protein mE‐L‐SNA‐induced lysis and completely retain the intact surface structures required for specific attachment to mammalian cells. EcN BGs were then loaded with the anticancer agent Epothilone B, which induces apoptosis via the mitochondrial pathway in HeLa cells (Zhu *et al.*, 2018). Due to the external immunologic properties of living bacteria, EcN BGs were used as candidate adjuvants. This was done by cell lysate‐based anticancer vaccination of a syngeneic murine lung carcinoma model (Kraśko *et al.*, 2017). These results indicate that EcN BGs are a promising drug delivery carrier for drug candidates in cancer therapy.

**Figure 2:**
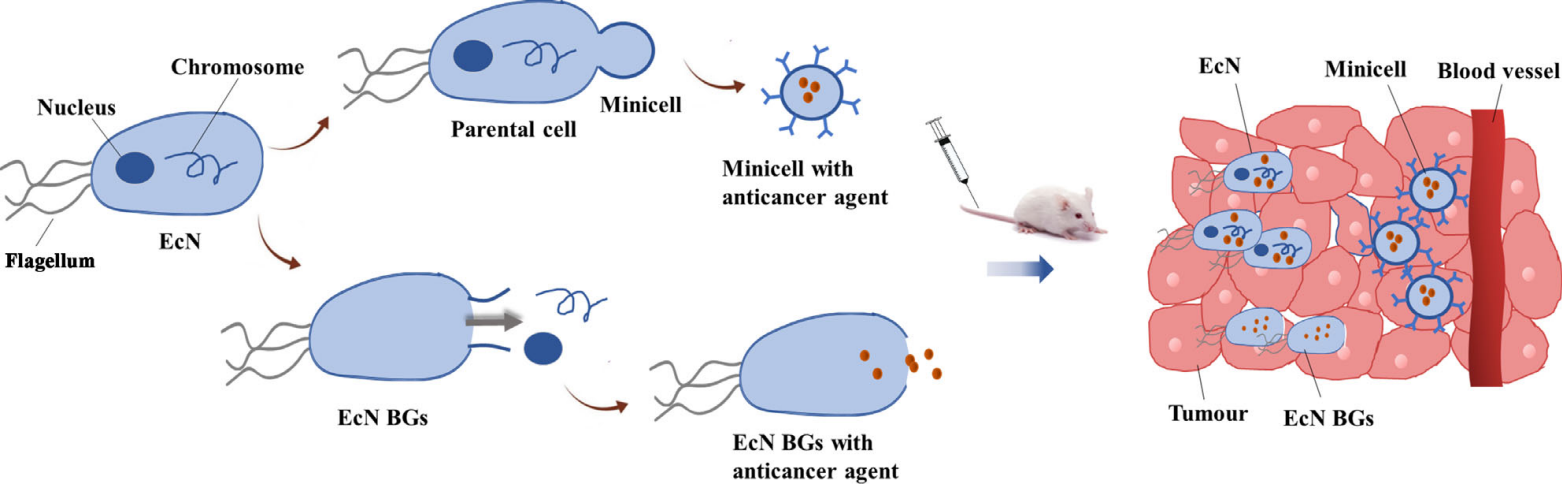
The payload delivery of EcN, EcN‐derived minicells and EcN BGs.

Overall, studies of EcN‐mediated tumour therapies have demonstrated that probiotic EcN can be engineered to safely and selectively deliver therapeutic payloads to the tumour microenvironment, and they can be used as an optimal chassis for living cancer therapeutics. The combination of EcN‐derived minicells with ligands against tumour‐associated markers has additional tumour‐targeting effects (Zhang *et al.*, 2018). EcN BGs exhibit excellent immunogenicity and can be used as candidate adjuvants for anticancer vaccination (Kraśko *et al.*, 2017). The choice of a suitable delivery system (EcN, EcN‐derived minicells or BGs shown in Fig. 2) depends on the experimental goals, all of which are aimed at improving the therapeutic index.

## Conclusions

A variety of studies have shown that EcN‐mediated tumour therapies can successfully regress tumours and promote survival in mice. This indicates that EcN is a versatile probiotic that can be adopted for additional clinical applications as living therapeutics. However, numerous challenges remain including genetic instability, targeting efficiency and limited drug production. All of these challenges may be addressed using powerful recombinant DNA and synthetic biology techniques. For example, genetic stability may be improved by incorporating engineered genes into the EcN’s genome and by limiting homologous recombination and horizontal gene transfer with CRISPR‐Cas9 technology. Targeting efficiency may be enhanced by the genetic manipulation of endogenous chemoreceptors. Gene expression is predominantly regulated at the level of transcription. The high‐level constitutive expression of heterologous proteins may lead to a metabolic burden to the bacterial vector, resulting in decreased stability and inefficient colonization. Thus, drug production could be manipulated by optimizing promoter strength, gene copy number, ribosome‐binding sites and bacterial metabolism. Due to its genetic flexibility, EcN may be rationally designed for clinical studies, resulting in a powerful weapon against cancer.

## Conflict of interest

None declared.
